# Chikungunya outbreak in a rural area of Western Cameroon in 2006: A retrospective serological and entomological survey

**DOI:** 10.1186/1756-0500-3-128

**Published:** 2010-05-05

**Authors:** Maurice Demanou, Christophe Antonio-Nkondjio, Emmanuel Ngapana, Dominique Rousset, Christophe Paupy, Jean-Claude Manuguerra, Hervé Zeller

**Affiliations:** 1 Laboratoire de Virologie, Centre Pasteur Cameroon, BP 1274, Yaoundé, Cameroon; 2 Organisation de Coordination pour la lutte contre les Endémies en Afrique Centrale (OCEAC), Yaoundé, Cameroun; 3 Comité National d'Epidémiologie du Cameroun, Yaoundé, Cameroun; 4 Institut de Recherche pour le Développement (IRD), UR016, OCEAC; 5 Institut Pasteur, Paris, France; 6 Centre national de référence des arbovirus et fièvres hémorragiques, Institut Pasteur, Lyon, France

## Abstract

**Background:**

Although arboviral infections including Chikungunya virus (CHIKV) are common in sub-Saharan Africa, data on their circulation and prevalence are poorly documented. In 2006, more than 400 cases of dengue-like fever were reported in Kumbo (Northwest Region of Cameroon). The aim of this study was to identify the aetiology of this fever and to define its extent in the area.

**Methods:**

We conducted a cross-sectional seroprevalence survey one year after clinical investigations to define the extent of the infection. An entomological survey consisted of the collection and identification of mosquito immature stages in water containers in or around human dwellings.

**Results:**

A total of 105 sera were obtained from volunteers and tested for CHIKV, O'Nyong-nyong virus (ONNV) and Dengue virus (DENV) specific IgM and IgG antibodies by enzyme-linked immunosorbent assays (ELISA). CHIKV infection was defined as the presence of IgM antibodies to CHIKV. There was serological evidence for recent Chikungunya infection, as 54 subjects (51.4%) had detectable IgM anti-CHIKV in their sera. Amongst these, 52 showed both anti-CHIKV IgM and IgG, and 2 (1.9%) had IgM anti-CHIKV in the absence of IgG. Isolated anti-CHIKV IgG positives were detected in 41 (39%) cases. No anti-ONNV and anti-DENV IgM antibodies were found amongst the sample tested. Out of 305 larvae collected in the different breeding sites, 87 developed to the adult stage; 56 (64.4%) were *Aedes africanus *and the remaining *Culex *spp.

**Conclusions:**

These findings suggest that the outbreak of febrile illness reported in three villages of Western Cameroon was due to CHIKV. The issue of a possible persistence of anti-CHIKV IgM antibodies is discussed. *Ae. africanus *which was found to be relatively abundant among the raffia palm bushes probably plays a role in the transmission of CHIKV along the chain of sylvatic/domestic mosquito species in this rural area. Particular attention should therefore be given to arbovirus infections in the Central African sub-region where these infections are becoming an emerging public health threat.

## Background

Chikungunya virus (CHIKV), is a member of the *Alphavirus *genus (*Togaviridae *family) and belongs to the Semliki virus antigenic complex comprising species with similar clinical manifestations and close antigenic patterns like CHIKV, O'Nyong-nyong virus (ONNV), Ross River virus and Mayaro virus [1,2].

CHIKV is now known to circulate in enzootic cycles throughout much of sub-Saharan Africa where it originated. In West and Central Africa, the disease is maintained in a sylvatic cycle involving wild non-human primates and forest-dwelling *Aedes *spp. mosquitoes. The virus has been isolated from sylvatic mosquito species in several countries including Senegal, Côte d'Ivoire, Central African Republic and South Africa. The mosquito species involved vary geographically and with ecological conditions; however, the major species involved in sylvatic cycles are *Ae. furcifer*, *Ae. taylori*, *Ae. luteocephalus*, *Ae. africanus *and *Ae. neoafricanus *[3-5]. In rural regions, outbreaks of human disease are occasionally detected when adequate laboratory diagnostics are implemented. These outbreaks tend to be of small scale and appear to be heavily dependent upon the sylvatic mosquito densities that increase with periods of heavy rainfall [6].

In Asian and African urban cycles, the disease is transmitted by *Ae. aegypti *and *Ae. albopictus *with humans and mosquitoes as the only hosts of the virus [7,8]. CHIKV has been responsible for many major outbreaks since its discovery in 1953 [9] but also has apparently disappeared for long periods during which it probably occurred only as sporadic or asymptomatic cases. From 2004, cases of CHIKV have risen meteorically in urban areas [10-13] leading to the recognition of CHIKV as a dangerous and important emerging arbovirus [14].

In Cameroon, cases of CHIKV were reported in 2006 in the two main cities of the country: Douala and Yaoundé [15]. That same year, in a rural locality in Kumbo (Bui division, Northwest Region of Cameroon), situated about 300 km north of Yaoundé, about 400 inhabitants of three neighbouring villages exhibited a dengue-like syndrome. From the clinical investigations carried out from October 31 to November 1, 2006 by the local health authorities, it appears that these clinical manifestations started in April 2006, at the beginning of the rainy season, becoming more serious in August. They were characterized by fever, chills, headache and muscle pains, combined with prolonged and severe joint pains. Unfortunately, this information reached the Ministry of Health (MOH) only in 2007 when a CHIKV epidemic was reported in Gabon, a neighbouring country [13]. The MOH then initiated field investigations so as to identify the aetiology of this fever and to define its extent in the area.

This paper describes the findings of the serological and entomological surveys conducted from November 18-26, 2007, at the beginning of the dry season in three villages of Kumbo. Results of such a study are invaluable for public health systems in Central African region where epidemics are frequently underreported or misreported.

## Methods

### Study site

Investigations were carried out in three villages, Ngehndzen, Ndzeru and Tasaï where clinical cases were registered during the outbreak in 2006.

**Figure 1:**
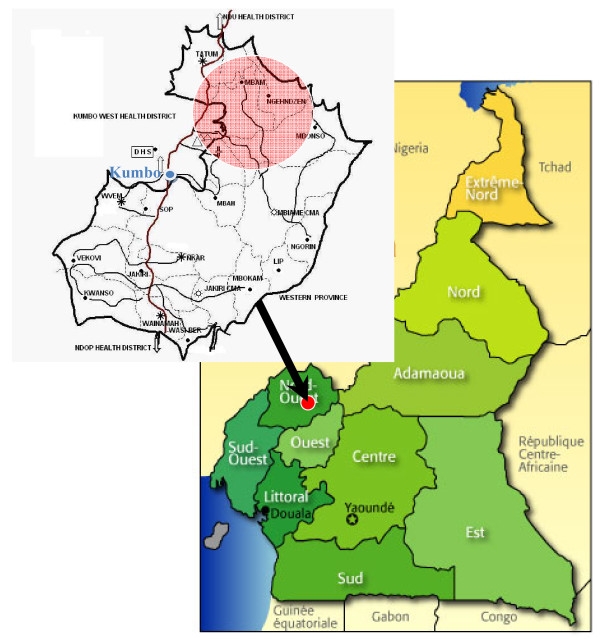
**Location of the study area in the Northwest Region of Cameroon**.

These villages belong to the Mbam health care area and are situated about 30 km north-east of the centre of Kumbo (Figure 1). They all lie on the slopes of mountains at about 1000 - 1500 m above sea level with very difficult accessibility. The vegetation is mainly of grass fields with few trees primarily distributed in lowlands. Raffia palm bushes are common in the area and are found along the valleys and streams. The main occupation of the villagers is subsistence farming. Main crops grown by villagers are vegetables, plantains, groundnuts, coco yams, coffee and maize. The climate in the area is equatorial with two seasons: a rainy season running from mid-March to mid-November and a dry season from mid-November to mid-March.

### Study design and data collection

We conducted a cross-sectional seroprevalence study one year after clinical investigations. A week before the investigation, a local health care worker went round the villages to make the population aware of the study. At our arrival, informed consent was obtained from volunteers gathered at the meeting points (health care centres and centres of villages) before blood collection. As the study was considered by the MOH as part of its public health response to the outbreak (letter number D112-212/NS/MSP/SG/CNEC/SP), no authorization was required from the national ethics committee.

During the entomological survey oral consent of householders was requested for inspection of their houses. Emphasis was put on households where cases of acute febrile syndrome were reported the previous year. Indoor and outdoor water storage (i.e. domestic), abandoned (i.e. peridomestic) containers, and natural receptacles and pools containing water were inspected in human dwellings and among raffia bushes for evidence of the presence of immature stages of *Aedes *spp mosquitoes. Larvae and pupae were collected and reared until the adult stage which was identified using morphological keys.

### Laboratory methods

Sera were separated from whole blood specimens and tested for IgM and IgG antibodies to CHIKV and ONNV in the Reference Centre for Arboviruses, Lyon (France), and antibodies to DENV in the laboratory of virology (Centre Pasteur Cameroon) using "in-house" techniques such as IgM capture enzyme immunoassay (MAC-ELISA) and ELISA IgG. For MAC-ELISA, IgM antibodies were captured with a goat anti-human IgM antibody (I2386, Sigma Laboratories). CHIKV, ONNV or DENV antigens were then added. Specific binding was demonstrated by using a CHIKV, ONNV or DENV mouse hyper immune ascitic fluid and a goat anti-mouse peroxidase-labelled conjugate (A0170, Sigma Laboratories). For the detection of IgG, microplates were coated with specific antigens (CHIKV, ONNV or DENV) and a specific binding was demonstrated by using a peroxidase-labelled goat anti-human IgG conjugate.

Sera were considered positive when the optical density of the assay exceeded the mean optical density of negative controls plus three standard deviations.

### Statistical analysis

Results of serological analysis were entered and analyzed using Epi Info 2002 software (Centre for Disease Control and Prevention). Age, sex, and locality distributions of the seropositive participants were compared. χ^2 ^was used for statistical testing and the level of statistical significance chosen for all analyses was p < 0.05.

## Results

A total of 105 persons (sex ratio m/f 0.44) were sampled for blood collection of whom 95 reported suffering clinical manifestations of a dengue-like syndrome during the outbreak period (about one year before this survey). The mean age was 50 years (minimum 8, maximum 81) with a standard deviation of 17.5. Most of the people examined were farmers (85.8%).

There was serological evidence for recent Chikungunya infection, as 54 subjects (51.4%) had detectable IgM anti-CHIKV in their sera (Table 1). Amongst these, 52 showed both anti-CHIKV IgM and IgG, and 2 (1.9%) had IgM anti-CHIKV in the absence of IgG. Isolated anti-CHIKV IgG positives were detected in 41 (39%) cases. There was no evidence for recent infection by ONNV because all the samples were seronegative for anti-ONNV IgM.

**Table 1:** Anti-CHIKV specific antibodies (IgM and IgG) detection by age and sex, in 105 people of the three villages surveyed in Kumbo, Cameroon, November 2007

	N	IgM positive (%)	IgG positive (%)
Age group (years)			
5-14	6	3 (50.0)	6 (100)
15-29	5	4 (80.0)	4 (80.0)
30-44	25	14 (56.0)	21 (84.0)
45-59	25	13 (52.0)	23 (92.0)
>= 60	44	20 (45.5)	41 (93.0)
Sex			
Females	73	38 (52.0)	66 (90.4)
Males	32	16(50.0)	29 (90.6)
Total	105	54 (51.4)	95 (90.5)

N, sample size

Nevertheless, all the 95 sera (90.5%) that were positive for anti-CHIKV IgG antibodies were also positive for anti-ONNV IgG antibodies due to the cross reactivity occurring between CHIKV and ONNV. No antibodies to DENV were detected.

IgM seropositivity to CHIKV was similar between females (52%) and males (50%) (Table 1). Because few participants between 5 and 29 years were enrolled in the survey, 5-14 and 15-29 year age groups were combined for statistical purposes; thus, anti-CHIKV IgM antibody prevalence did not vary significantly across different age strata, neither did anti-CHIKV IgG antibodies.

Seropositivity to CHIKV was similar in the different villages (Table 2).

**Table 2:** Anti-CHIKV specific antibodies (IgM and IgG) detection in each of the three villages surveyed in Kumbo, Cameroon, November 2007

Village	N	IgM positive (%) [95% CI]	IgG positive (%) [95% CI]
Ndzeru	41	20 (48.8) [33.5-64.1]	38 (92.7) [84.6-100]
Ngehndzen	36	20 (55.6) [39.2-71.9]	35 (97.2) [91.8-100]
Tasaï	28	14 (50.0) [31.5-68.5]	22 (78.6) [63.1-94.1]
Total	105	54 (51.4) [41.9-61.0]	95 (90.5) [84.8-96.1]

N, sample size

During the entomological survey 78 householders gave their oral consent for inspection of their houses. Most of them reported suffering the acute febrile syndrome the previous year. Eighteen to 30 households of 3 to 6 houses were surveyed in each village. All water containers, in or around human dwellings were inspected for the presence of mosquito larvae. Culicine larvae were found in 4 peridomestic breeding sites (old buckets, puddles) outside human dwellings and in 3 locations situated in raffia bushes where water had collected. Out of 305 larvae collected in these different breeding sites, 87 developed to the adult stage; 56 (64.4%) were *Aedes africanus *and the remaining *Culex *spp. Moreover, a number of adults of *Ae. africanus *mosquitoes were identified biting in raffia bushes during the day and this was consistent with complaints by the local populations of a high burden of mosquito bites in this particular biotope. Although only 3 breeding sites were found with culicine larvae in this environment, it was evident that places where water collected among raffia bushes probably represented important breeding places for *Ae. africanus *immature stages.

## Discussion

The prevalence of anti-CHIKV IgM antibodies one year after the outbreak was high and similar to data recorded during previous outbreaks of CHIKV [16,17]. Nevertheless, such a high prevalence was unexpected due to the delay between the outbreak and our study but was in accordance with the occurrence of sporadic cases of CHIKV infection in the area. Generally, high IgM antibody prevalences are recorded during the peak incidence of an outbreak as observed in the Comoro Island [17], but tend to decrease with time as reported by Sergon et al. on the Kenyan Island of Lamu [18] and Thonnon et al. [16] in Senegal during a yellow fever outbreak survey.

Although we did not collect blood samples from unaffected areas (control) for comparison purposes, the possibility of the persistence of IgM anti-CHIKV for one year cannot be ruled out. This apparent persistence of anti-CHIKV IgM is consistent with the observations of both Fourie & Morrison and Gauzere [19,20] who described such persistence of IgM antibodies for up to 12 months in patients suffering from polyarthritis or rheumatoid arthritis after CHIKV infection; and with those of Olivier et al. [21] who recorded 75 and 42% of anti-CHIKV IgM positives, respectively after 7 and 10 months, in French patients with imported CHIKV between 2005 and 2007. These observations closely match the situation in our study where many people were still complaining of recurrent arthralgia (joint pains). However, as we did not include a control village in our study, we could not totally discard the possibility of endemic circulation of the virus in the area. The fact that 39% of the samples were only IgG anti-CHIKV antibody positive could also be a result of the herd immunity from previous exposure to the virus as observed in healthy rural populations of Southern Cameroon by Kuniholm et al. [22].

Although arboviral infections including CHIKV are common in sub-Saharan Africa [1,13,16,23-26], data on their circulation and prevalence in Central Africa are poorly documented due to confusion between arboviral infection and hyperendemic *Plasmodium falciparum *infection and/or lack of diagnostic tools in local health care centres. Thus only a few sporadic cases of arboviral infections have been reported in recent years across Cameroon [15,22,27]. However, and despite evidence of the circulation of CHIKV in the country, this study to the best of our knowledge is the first report of an important rural epidemic of CHIKV in Cameroon.

The outbreak affected equally the three villages in Kumbo (Ngehndzen, Ndzeru and Tasaï), and both sexes, and all age groups. Despite the over-representation of women (sex-ratio 0.44) among the persons sampled during this survey, both sexes exhibited the same seropositivity rates. The possible explanation to this is that women, men and children are equally exposed to *Aedes *spp bites either in their dwellings or in the raffia bushes where they fetch firewood (women), collect palm wine (men) and water (children).

Despite the fact that the extensive serologic overlap occurring between CHIKV and ONNV precludes any definitive interpretation of the results as to which virus may have been responsible for the production of the IgG antibodies in 90.5% of the sera [28], the overall optical density of the CHIKV ELISA was three to eleven times more than that of ONNV (data not shown). This result is consistent with the comparative studies between CHIKV and ONNV supporting the one-way antigenic relationship between CHIKV and ONNV [29,30].

As the CHIKV outbreak in Kumbo and Yaoundé/Douala occurred during the same period, it is possible that the same virus strain was implicated. It has been shown that the Cameroon strain is highly similar to the CHIKV strain reported in the sub-region including Democratic Republic of Congo and Gabon [15]. However, the occurrence during these recent years of epidemic or sporadic cases highlights the possible mutation of the virus, with the introduction of a new and competent vector *Ae. albopictus *into Africa [31]. Indeed isolates from the Indian Ocean (La Reunion, etc.) were found to carry a mutation at residue 226 of the membrane fusion glycoprotein E1 (E1-A226 V); and it was demonstrated that such a mutation provided a selective advantage for the replication and transmission of CHIKV by this mosquito [32,33]. *Ae. albopictus *was identified as the primary vector in the Chikungunya outbreak in Libreville (Gabon) [34,35] and was suspected in the transmission of CHIKV in Yaoundé and Douala [15]. This invasive species burst into the African continent in the early 1990s and is now present in most Central African countries [36-38]. However no *Ae. albopictus *or *Ae. aegypti*, were found during this survey probably because the study took place during the dry season which is probably not suitable for their emergence. Moreover, these two species were described in the North-West regional capital (about a hundred km to the south) during the rainy season [38].

Although we did not search for the virus in mosquitoes collected, *Ae. africanus *could at least be involved in the transmission of CHIKV down the chain of sylvatic/domestic mosquito species in this area. It is possible that by entering areas like raffia bushes, and so being in contact with the sylvatic *Ae. Africanus*, humans might have become infected therefore serving as their incidental hosts. These humans might have then provided a source of the virus to infect potential peridomestic mosquitoes (although this was not found during our survey), which then became involved in the transmission cycle of the virus. CHIKV is known to circulate in a natural cycle amongst forest-associated simians and sylvatic mosquito species in West and Central African jungles, spasmodically causing outbreaks of varying size and intensity amongst the local populations living near the jungles [39,40].

## Conclusions

Between April and July 2006, CHIKV was identified during an outbreak of a febrile syndrome in Yaoundé and Douala [15]. In the mean time, in a rural area of Western Cameroon, several people also suffered from acute febrile illness. Although investigations were carried out one year after the latter outbreak, these findings suggest a recent circulation of CHIKV in three villages of Kumbo (Western Cameroon). *Ae. africanus *which was found to be relatively abundant in the raffia bushes could be involved in the transmission of CHIKV along the chain of sylvatic/domestic mosquito species in this rural area via humans and simians that come into contact with the virus either in the gallery forest or raffia bushes surrounding the villages.

This study, alongside previous ones, provides further evidence of the circulation of the CHIKV in the Cameroon forest region. Despite the co-endemicity of malaria and arbovirus infections in the Central African sub-region CHIKV infection is an emerging public health threat which should be given particular attention. In the absence of a vaccine, more effort should be put into prevention by involving the local population in vector control activities.

## Competing interests

The authors declare that they have no competing interests.

## Authors' contributions

MD and EN conceived and designed the study. CAN carried out entomologic investigations. HZ performed CHIKV serologic assays. MD and CP analysed the data. MD wrote the initial draft and CAN, DR and JCM revised the manuscript. All authors read and approved the final manuscript.

## Authors' information

MD is a young virologist in charge of the newly created Arbovirus and Haemorrhagic Fever virus branch in the Laboratory of Virology at Centre Pasteur Cameroon. Since 2006, his research interests have focused on diagnosis and epidemiology of arbovirus diseases (dengue, chikungunya, yellow fever, etc.).
